# An Approach for Brain-Controlled Prostheses Based on a Facial Expression Paradigm

**DOI:** 10.3389/fnins.2018.00943

**Published:** 2018-12-18

**Authors:** Rui Li, Xiaodong Zhang, Zhufeng Lu, Chang Liu, Hanzhe Li, Weihua Sheng, Randolph Odekhe

**Affiliations:** ^1^Shaanxi Key Laboratory of Intelligent Robot, Xi'an Jiaotong University, Xi'an, China; ^2^School of Electrical and Computer Engineering, Oklahoma State University, Stillwater, OK, United States; ^3^Shenzhen Academy of Robotics, Shenzhen, China

**Keywords:** facial expressions, electroencephalography (EEG), brain computer interface (BCI), brain-controlled prosthesis, the motor cortex, the prefrontal cortex

## Abstract

One of the most exciting areas of rehabilitation research is brain-controlled prostheses, which translate electroencephalography (EEG) signals into control commands that operate prostheses. However, the existing brain-control methods have an obstacle between the selection of brain computer interface (BCI) and its performance. In this paper, a novel BCI system based on a facial expression paradigm is proposed to control prostheses that uses the characteristics of theta and alpha rhythms of the prefrontal and motor cortices. A portable brain-controlled prosthesis system was constructed to validate the feasibility of the facial-expression-based BCI (FE-BCI) system. Four types of facial expressions were used in this study. An effective filtering algorithm based on noise-assisted multivariate empirical mode decomposition (NA-MEMD) and sample entropy (SampEn) was used to remove electromyography (EMG) artifacts. A wavelet transform (WT) was applied to calculate the feature set, and a back propagation neural network (BPNN) was employed as a classifier. To prove the effectiveness of the FE-BCI system for prosthesis control, 18 subjects were involved in both offline and online experiments. The grand average accuracy over 18 subjects was 81.31 ± 5.82% during the online experiment. The experimental results indicated that the proposed FE-BCI system achieved good performance and can be efficiently applied for prosthesis control.

## Introduction

With the increase in the number of disabled persons with amputations or spinal cord injuries, many studies have focused on the development of prosthetic technology to restore lost motion function (Ziegler-Graham et al., 2008). Research on prosthesis control strategies is expected to enable patients who can use a prosthesis as an assistive device to realize their routine activities (Makowski et al., 2014). Several types of prosthesis have been developed, ranging from passive cosmetic prostheses to body-powered limbs, from EMG-based prostheses to EEG-based prostheses (Lee et al., 2014). The earliest prostheses were passive cosmetic devices, which can only help a person seem less awkward in social situations but not change posture (Cordella et al., 2016). However, body-powered prostheses gradually replaced the passive cosmetic prostheses due to their simple design and effectiveness. The shortcoming of these types of prosthesis is that they can only control one joint at a time by mechanical linkage (Kistenberg Robert, 2014). With developments in prosthetic technology, considerable attention has been focused on biological signal control strategies because these signals can represent a person's own intention. Due to the distinct neural information of EMG, these signals play an important role in prosthesis control technology (Lobo-Prat et al., 2014; Madusanka et al., 2015). Two types of EMG prostheses, the I-limb system and the ProDigits system, are commercially available due to their characteristics such as high recognition accuracy and minimal complexity (Pan et al., 2014). However, EMG-controlled prostheses can only be effective if they satisfy two premises. Firstly, the amputees could voluntarily activate repeatable and distinct EMG signal patterns for different motor tasks associated with their limb movements. Secondly, the amputees have enough residual muscles to provide EMG signals with rich set of neural information for accurate limb movement intents decoding (Kuiken et al., 2009; Al-Angari et al., 2016).

Since EEG signals are independent of residual muscles and contain high-quality neural information on an individual's intentions, developments in BCI systems are designed to control prostheses using a subject's thoughts alone (Donchin et al., 2000; Mcmullen et al., 2014; Stan et al., 2015; Vidaurre et al., 2016), which can be divided into spontaneous BCIs [event-related (de)synchronization (ERD/ERS)] and evoked BCIs [steady-state visual evoked potential (SSVEP), P300 potential, etc.] (Pfurtscheller et al., 2000b; He et al., 2016). Several efforts have been exploited that use BCIs to control prosthesis, and the main system include motor-imagery-based BCIs (MI-BCIs) and SSVEP-based BCIs (SSVEP-BCIs) (Acharya et al., 2010; Wang and Veluvolu, 2017). The first report to use a MI-BCI system to control a prosthesis was presented by the Graz University of Technology. After several months of training, their accuracy was close to 90% (Pfurtscheller et al., 2000a). Another study showed how monkeys could use their motor cortical activity to control a mechanized arm in a self-feeding task. This study was the first to add physical interactions between a 5 degrees of freedom (5-DOFs) robotic arm and physical objects (Velliste et al., 2008). Recently, a MI based brain-controlled prosthesis using multiple controlled tasks has been reported. The MI-BCI was associated with multiple classes of imagined upper limb movements collected from 64-channel EEG signals, and it has showed good performance (Samuel et al., 2017b). Although this kind of BCI system has some advantages, such as stable and rapid responses, the long duration of training and variability between different users limits their further study.

Another widely adopted BCI system is the SSVEP-BCI, which responds to visual stimulation (Vialatte et al., 2010). The most common paradigm used in this area is flashing light patterns, which have been successfully used to control a 2-DOFs hand orthotic using only 2 EEG signals. The mean accuracy of this method can reach 85% without training (Pfurtscheller et al., 2010). In another development, a scene graph SSVEP-BCI system for control of a 2-DOFs prosthesis using 2 EEG channels recording from the occipital cortex has been reported. The merits of this method are the high information transfer rate (IRT), high recognition accuracy and lack of training time required; however, this system relies entirely on a stimulator (Xie et al., 2012; Chen et al., 2014). Moreover, a long stimulation time may easily lead to epileptic seizures.

Under all obstacles above, there remains motivation for finding a novel BCI method. Providing an alternative BCI system to overcome the limitations between high accuracy and independence is necessary. Recently, another kind of novel BCI system, which is called a FE-BCI system, has been developed. Most researchers have focused on face-based video or face-based image induced systems to develop a FE-BCI system (Kashihara, 2014; Daly et al., 2016; Toth and Arvaneh, 2017). Jin and colleagues introduced a visual stimulus pattern based on the images of facial expression, the presentation of images of face could successfully evoke ERPs (Jin et al., 2014). However, only a few studies have used real facial expressions. Chin and colleagues reported a technology that classified facial expressions based on EEG and EMG signals using the Filter Bank Common Spatial Pattern (FBCSP) algorithm (Chin et al., 2008). However, this system is not a complete BCI system. As EEG signals are very sensitive to EMG signals, the contributions of each kind of signal was unknown. Additionally, the location of the most contributive channels was not investigated.

In this study, we hypothesized that the responses from the prefrontal and motor cortices contain important information relevant to different facial expressions. To verify the feasibility of the proposed paradigm, a FE-BCI system was implemented using four facial expressions to control a 2-DOFs prosthesis. The organization of this paper is as follows. Section Materials and Methods addresses methodology, including the mechanisms of facial expression, the brain-controlled prosthesis based on a FE-BCI system, the experimental setup and data analysis. Section Results describes the brain response experimental results as well as corresponding accuracies. The discussion and conclusion are stated in Sections Discussion and Conclusions, respectively.

## Materials and Methods

The neural pathway mechanisms of different EEG signals provide the theoretical foundation for a BCI system. In this section, we described the biological mechanisms of facial expression formation and the construction of a brain-controlled prosthesis system. Moreover, the experimental setup and the EEG signal analysis algorithm are also systematically investigated.

### Mechanisms of Facial Expression

A new field of neurophysiology is that of affective computing, which integrates systems that analyze and process human emotions (Marinkovic and Halgren, 1998; Etkin et al., 2011). One of the most fundamental features that depicts human emotions is facial expression, which synthesizes several basic emotions (Keltner et al., 2003; Kilts et al., 2003). Multiple factors appear to contribute the mechanisms of human facial expressions, including brain responses, nervous system transmissions, motor neuron activity generation, facial nerve transmissions and realizations, and facial muscle movements (Mandal and Awasthi, 2015). Earl‘s group and other similar studies have extensively reported that several of the critical abilities of the prefrontal cortex are related to cognitive control, goal-directed behavior and facial expression (Marinkovic et al., 2000; Miller and Cohen, 2001; Gray et al., 2002; Lisetti and Schiano, 2008). The role of facial expressions in physiological emotional processes and conscious emotional experiences has inspired considerable discussion. One of the most distinguished result from the previous studies was that the change of facial expression could result in the corresponding brain activity over the prefrontal cortex, especially in alpha and theta band (Friedman and Thayer, 1991; Coan et al., 2001). Hence, facial expression is an effective method to intensified emotions-specific responses over prefrontal.

Furthermore, a series of studies by Ross and Guillermo demonstrated that brain responses to facial expressions are also seen in the motor cortex (Paradiso et al., 2005; Ross et al., 2016). Thus, the brain responses over the motor cortex might provide a possible link between this area and the representation of facial expression. Facial expressions can generally be divided into the upper facial expressions and the lower facial expressions. The upper facial expressions mainly concern with the expressions from brows and eyes. The lower facial expressions are related to the expressions around cheek and mouth. Interestingly, researchers found that the upper facial expressions have its responses reflected on the prefrontal cortex and motor cortex, while the lower facial expressions have more distinguished activity on motor cortex.

Considering the mechanisms of facial expression and the goal of our study, four facial expressions [Raising Brow (RB), Furrowing Brow (FB), Left Smirking (LS) and Right Smirking (RS)] were selected for the proposed FE-BCI system. The simplicity, repeatability and the distinct differences of the involved brain cortices among facial expressions were taken into account. In relation to the emotional experience, these expressions were chosen because they convey typical emotions (e.g., Raising Brow is accompanied by shock emotions). Meanwhile, considering the responses over the motor cortex, RB and FB as two general upper facial expressions were selected due to its simplicity and repeatability. Also, LS and RS one-sided lower facial expressions were selected to enlarge the different response of the motor cortex. In a nutshell, RB, FB, RS, and LS were used in the FE-BCI system and the brain responses from the prefrontal and motor cortices serves as the regions of interest to classify the different facial expressions.

### Description of Brain-Controlled Prosthesis System

**Figure 1:**
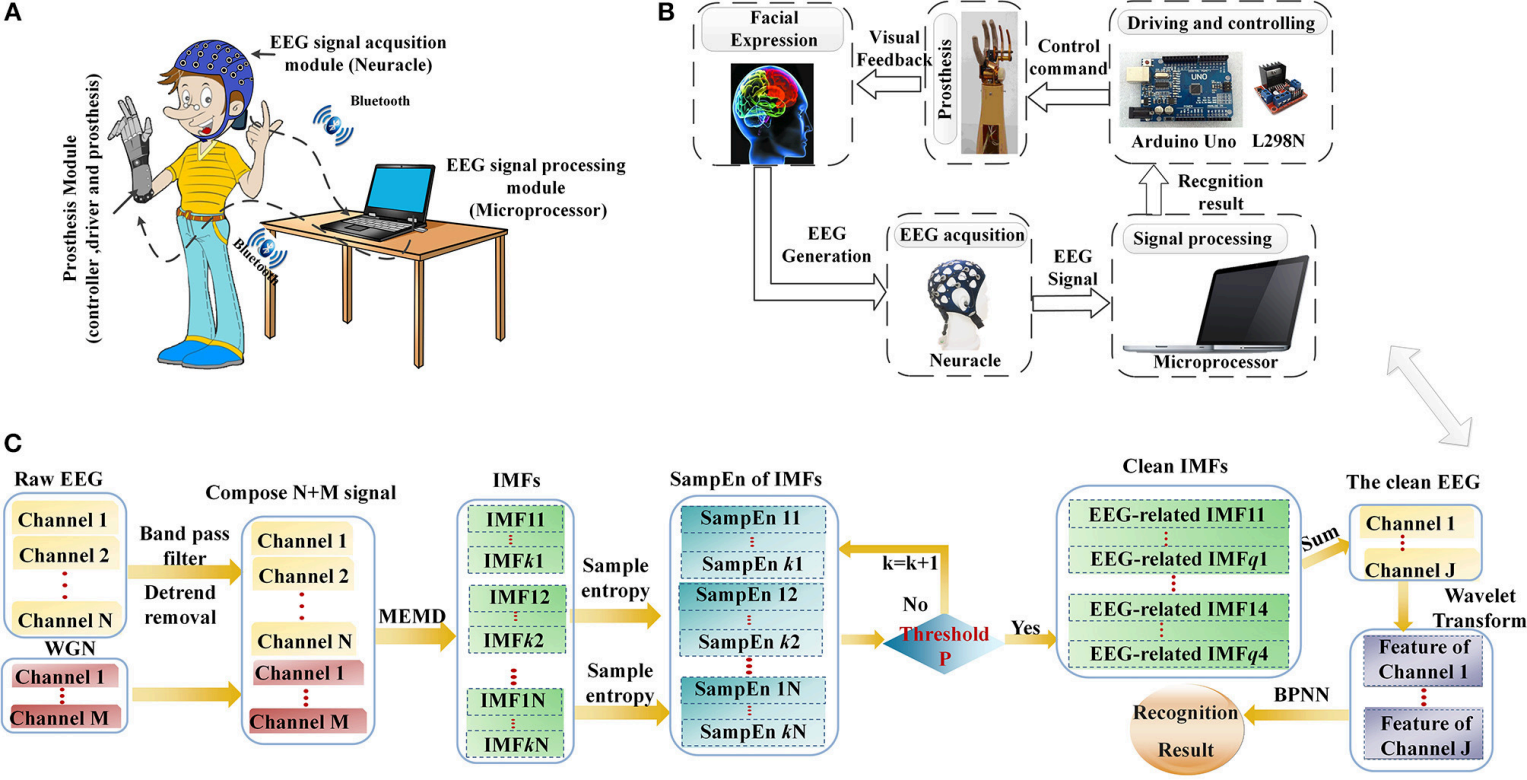
The brain-controlled prosthesis system based on facial expressions and its schematic description. **(A)** The 2-DOFs brain-controlled prosthesis system. **(B)** A schematic of the FE-BCI system-controlled prosthesis. **(C)** Flowchart of EEG signal processing.

Based on previous experiences and the performance criteria for prosthesis control (He et al., 2016; Minguillon et al., 2017), the system used in this study was composed of three modules: EEG signal acquisition, EEG signal processing and the prosthesis. An 8-channel wireless Neuracle manufactured by Neuracle Technology Co., Ltd, was selected as the EEG signal-acquisition module, and a microprocessor with Intel (R) Core (TM) i5-5600 CPU was employed as the EEG signal-processing module. The prosthesis module was custom-made by Danyang Artificial Limb Co., Ltd. and integrated an Arduino Uno controller, a L298N motor driver, 2-DOFs prosthesis with wrist and finger joints and a Bluetooth device. An overview of the system is illustrated in Figure 1A.

The prosthesis-control strategy and corresponding schematic of EEG signal analysis are shown in Figures 1B,C. When the system working, each facial expression task was observed for 4 s, and a rest session of 2 s was introduced between two consecutive tasks. Neuracle recorded EEG signals from the prefrontal and motor cortices during the task, which were simultaneously transferred to the microprocessor by Bluetooth. Then, the microprocessor processed the EEG signals in two steps. The first step was EMG artifacts removal, which was calculated by NA-MEMD and SampEn algorithms. After EMG artifacts removal, the feature set of the resulting signal was extracted by WT, and BPNN was used to discriminate the subject's intention. Finally, the recognition result was translated into a control command to actuate the driving and controlling devices, and then the prosthesis was operated based on the subject's intention. The delay that occurred while sending the control command from the microprocessor to controller was 200 ms. More details of EEG data analysis can be found in Section Data processing.

### Subjects and Data Acquisition

Eighteen healthy subjects (22–30 years of age, 15 males and 3 females) participated in this study, without any experience of the proposed FE-BCI system. None of them received any training before the experiments. Written informed consent was obtained from each subject before the experiment. The Institutional Review Board of Xi'an Jiaotong University approved the proposed experiment, and all experiments were conducted in accordance with the Declaration of Helsinki. The detail method of sample size estimation can be found in section Statistical analysis.

**Figure 2:**
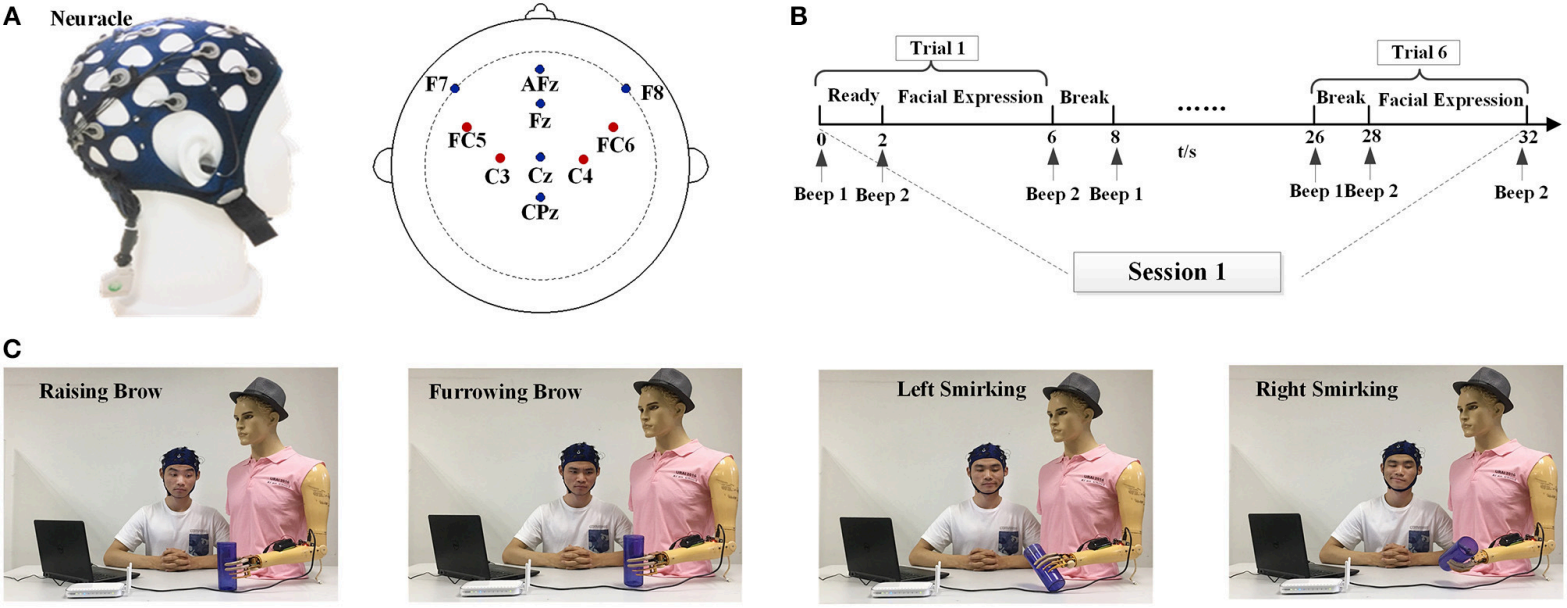
Experimental setup and task description. **(A)** The Neuracle device and its electrode placements. **(B)** Overview of the time series of one session. **(C)** The experiment scene with one subject (S3) to illustrate the four facial expression tasks used for prosthesis movements. Raising Brow corresponds to hand opened, Furrowing Brow corresponds to hand closed, Left Smirking corresponds to wrist rotation to the right, and Right Smirking corresponds to wrist rotation to the left. Written informed consent for the publication of identifying images was obtained from the subject (S3).

EEG signals were acquired using Neuracle (Figure 2A) at sampling rate of 1,000 Hz. The Neuracle has 8 EEG channels and 2 references channels that collect EEG signals. The channel distributions are based on the international 10-20 electrode location system. According to the neurophysiological mechanisms of facial expressions and reduce the unavoidable EMG artifacts over the prefrontal and motor cortices, four electrodes were placed on FC5, FC6, C3, and C4, which are shown in Figure 2A. The electrodes AFz and CPz served as a reference and ground by the previous literature (Yao, 2001). The impedances for all electrodes were maintained below 5 kΩ. All online and offline data analyses were performed after resampling to 250 Hz. After this, a Butterworth bandpass filter was used to filter EEG data into 3–30 Hz frequency bands.

### Experimental Procedure

In the experiment, subjects were instructed to sit behind a table and avoid body movements during the experiment; the seat was placed 20~50 cm away from the desk. All subjects were instructed to perform both offline and online experiments. There were 10 sessions for each experiment, and each session consisted of 6 trials. In each trial, a beep will alert the subject that the experiment is just about to begin; the subjects have 2 s to prepare and then instructed to perform one of four facial expressions within for 4 s. To avoid mental fatigue, there were 2-s breaks between every 2 trials and a 5-min intermission between every 2 sessions. The time series of one session is shown in Figure 2B.

Figure 2C depicts the experiment scenes from one subject (S3). During the offline stage, the subjects were instructed to repeat the RB, FB, RS, and LS movements. Each subject was asked to perform 10 repetitions of each facial expression. After the best feature sets had been determined for each facial expression of each subject, an online task to imitate drinking water was conducted.

In the online stage, each subject performed 10 sessions with the same time series as the offline experiment. Due to grasp pattern being one of the most important functional motion for human hand, it can help disabled person to perform more complicate activities without requiring additional support, such as holding a bag, drinking, eating, among other. Thus, four kind of actions were selected; hand opening, hand closing, wrist rotating right and wrist rotating left, respectively. In each session of online experiment, the subjects had to finish a complete prosthesis movement that imitated the process of drinking water by a disabled person. The detailed procedure is as follows:

(1) Opening the prosthesis to prepare grasping cup.
(2) Closing the prosthesis to grasp the cup.
(3) Wrist rotating to the right direction. It means wrist rotation toward subject to drink water.
(4) Wrist rotating to the left direction. It means wrist rotation away from subject to prepare to put down the cup.
(5) Putting down the cup on the table and then open the prosthesis
(6) Closing the prosthesis to the initial state.

Four different facial expressions RB, FB, RS, and LS were performed to control hand opening, hand closing, wrist rotating right and wrist rotating left, respectively. During the online test, distinct results were generated every 0.5 s after the initial 2.5 s of each trial. Once the control command was detected, the classifier would not work until next trial begin to ensure the specific gesture of prosthesis finished.

### Data Processing

#### Artifact Removal

EMG artifacts in EEG signals may inevitably result misleading in signal detection. Many algorithms are used for EMG artifacts removal, among them, Independent Component Analysis (ICA) was the most-commonly used method in the BCI system. However, with the necessary assumption of multichannel condition, this technique is only effective when a sufficient number of channels are available (Boscolo et al., 2004). To overcome these obstacles, NA-MEMD has been developed with relatively good performance in terms of EMG artifacts removal in few-channel EEG signals due to its highly localized time-frequency representations and self-adaptation characteristics (Rehman and Mandic, 2009; Teng et al., 2014; Chen et al., 2017, 2018). Moreover, SampEn is an effective way to identify the complexity of different biological signals (Richman and Moorman, 2000; Liu et al., 2017). It is well known that the randomness of EMG signal is much stronger than that of EEG signal in the same condition, so the entropy of EMG signal will be larger than that of EEG signal. Therefore, an appropriate threshold of sample entropy can be determined to distinguish the EMG artifact. Taking the above properties into account, NA-MEMD combined with SampEn was used to reduce EMG artifacts in the FE-BCI system.

NA-MEMD simultaneously decomposes multichannel data, ensuring the better alignment of corresponding Intrinsic mode functions (IMFs) from different channels, which will benefit the specific feature extraction of EEG signals. Since the quasi-dyadic filter bank properties of MEMD on white Gaussian noise (WGN) and the broadband characteristic of white noise, IMFs corresponding to the original signal can exhibits a quasi-dyadic structure enforced by the extra noisy channels and thus, reduces the mode mixing problem (Ur Rehman and Mandic, 2011). Given that *y* (*t*) consists of the *N* channel EEG signals and *M* channel WGN with the same length, which is represented by:

(1) $$\begin{array}{r} \text{y}\left(t\right)={\left\{{\text{y}}^{1}\left(t\right),{\text{y}}^{2}\left(t\right)\ldots \ldots {\text{y}}^{\text{n}}\left(t\right)\right\}}_{\text{t}=1}^{\text{T}},\left(n=\text{1,2}\ldots \ldots M+N,t=1,2\ldots \ldots T\right) \end{array}$$

where *T* is the number of temporal samples, *n* is the total number of channels.

Given that *y* (*t*) can be decomposed into *J* scales of IMF by NA-MEMD, $y\left(t\right)=\sum_{j=1}^{J}{\text{h}}_{j}\left(t\right)+r\left(t\right)$, where *h*_*j*_ (*t*) is the *j*_*th*_ IMF of *y* (*t*) and *r* (*t*) is the residual components. The detailed steps of NA-MEMD are given as follows:

(1) Hammersley sequences were used to generate a suitable set of direction vectors $X^{\theta_{k}}$ on an (*n*-1) sphere, that is:
  (2) $$\begin{array}{r} X^{\theta_{k}}=\left[x_{1}^{k},x_{2}^{k},\ldots x_{n}^{k}\right] \end{array}$$
  Where $\theta^{k}=[\theta_{1}{}^{k},\theta_{2}{}^{k},...,\theta_{n-1}^{k}]$ is the direction angles corresponding to the direction vectors.
(2) The projection *P*^θ*k*^ of the input signal *y* (*t*) along the direction vector *X*^θ*k*^, for all *k*, was calculated; *P*^θ*k*^ is denoted by:
  (3) $$\begin{array}{r} {\left\{P^{\theta k}\left(t\right)\right\}}_{k=1}^{K}\;\left(k=1,2,\ldots \ldots ,K\right) \end{array}$$
  where *K* is the total number of direction vectors.
(3) All the time instants $t_{i}^{\theta^{k}}$ were calculated, corresponding to the maxima of the set of projected signals${\left\{P^{\theta k}\left(t\right)\right\}}_{k=1}^{K}$, where *i* denotes the maximal time point.
(4) $\left[t_{i}^{\theta^{k}},y\left(t_{i}^{\theta^{k}}\right)\right]$ was interpolated for all *k* to obtain the multivariate envelope curves${\left\{e^{\theta^{k}}\right\}}_{k=1}^{K}$.
(5) The average envelope curves were computed for all *k*, which were calculated by
  (4) $$\begin{array}{r} m\left(t\right)=1/K\sum_{k=1}^{K}e^{\theta^{k}}\left(t\right) \end{array}$$
(6) The detail component *h*_*j*_ (*t*) was subtracted from the input signal *y* (*t*), which is represented by:
  (5) $$\begin{array}{r} h_{j}\left(t\right)=y\left(t\right)-m\left(t\right)\;\left(j=1,2,\ldots ,J\right) \end{array}$$
(7) If the detail component *h*_*j*_ (*t)* satisfied the stop criterion for an IMF, the above steps were repeated until residual components *r*_*j*_ (*t*) satisfied the stop criteria. Otherwise, steps (2–6) were repeated until all projected signals satisfied the stop criterion.

To further identify the information for each IMF, SampEn is used as a criterion to select significant IMFs. SampEn is widely used to detect artifacts because of its ability to detect the complexity of changes in brain activity (Mahajan and Morshed, 2015; Al-Qazzaz et al., 2017; Cuesta-Frau et al., 2017). SampEn is calculated as follows:

(6) $$\begin{array}{r} SampEn\left(m,r,N\right)=-\ln\;\left[\frac{A^{m}\left(r\right)}{B^{m}\left(r\right)}\right] \end{array}$$

where *N* is the length of the IMF, *m* is constant, and *r* is tolerance. In this study, *m* = 2, *r* = 0.2^*^std, where *std* is the standard deviation of the data.

Considering previous literature and experimental experiences, we set a specific threshold of 0.45 (Friesen et al., 1990; Liu et al., 2017). If SampEn exceeded the threshold, the corresponding IMF was discarded as an EMG artifact of the EEG signals. Finally, the clean EEG signals were reconstructed by summing the EEG-related IMFs.

#### Feature Extraction

To detect the various brain responses induced by the facial expression tasks, we computed feature sets using the WT algorithm. This algorithm decomposes EEG signals into a set of basic functions that combine time and frequency characteristics. In this paper, the energy and variances of the wavelet coefficients of the alpha and theta rhythms from each facial expression served as the feature set of the signal.

**Figure 3:**
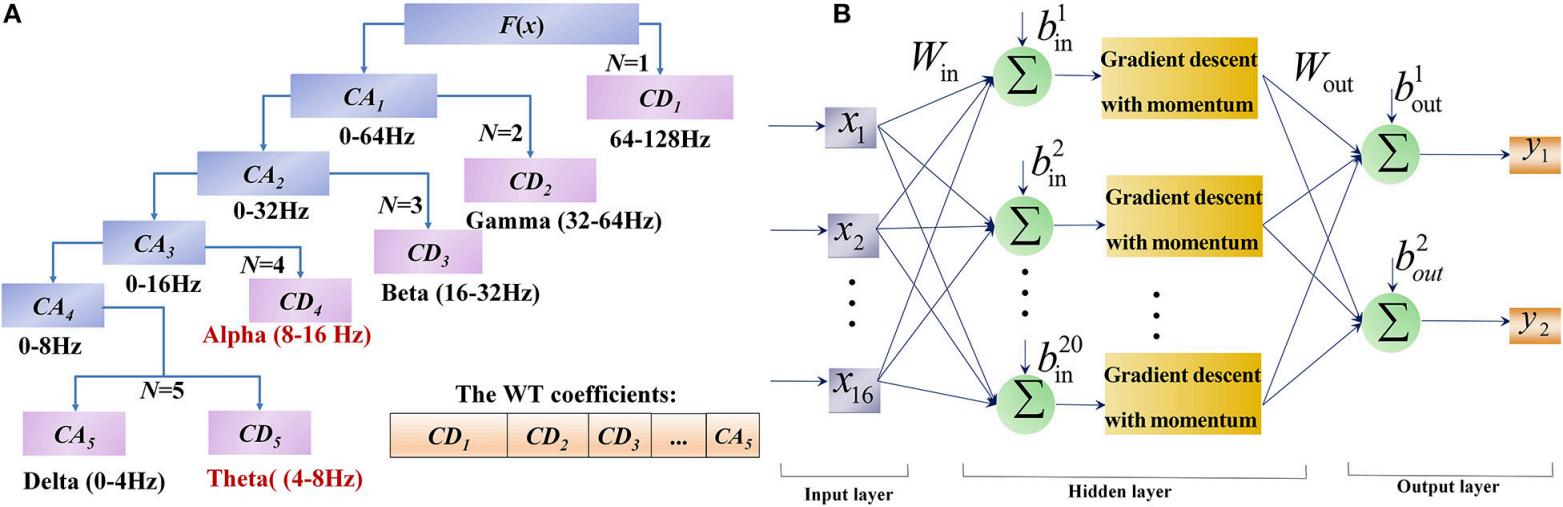
The flowcharts of WT and BPNN in FE-BCI system. **(A)** The flowchart of WT by 5 level decomposition. **(B)** The flowchart of BPNN with 3 layers and 20 nodes of hidden layer.

Considering the orthogonally, support set, symmetry, regularity and vanishing moment order, the db-3 wavelet was used as the WT basis function, and the decomposition level was set at 5 in this study. The procedure of WT decomposition is shown in Figure 3A. The EEG signal was decomposed into scales with different time and frequency resolution. After 5 series of wavelet decomposition, the EEG signal was separated into delta (0~4 Hz), theta (4~8 Hz), alpha (8~16 Hz), beta (16~32 Hz), gamma (>32 Hz) rhythms and the resulting WT coefficients could be further applied as signal features for its application (Wang et al., 2013). Since brain activity in the prefrontal and motor cortices are usually affected by different facial expressions, the feature sets of the synchronous brain responses induced by facial expressions include two statistics:

(1) Energy of the wavelet coefficients
  (7) $$P\alpha_{\text{j}}=\sum_{i=1}^{n}y_{i}^{2},P\theta_{j}=\sum_{i=1}^{n}y_{i}^{2}\;(j=1,2,3,4)$$
(2) Variance of the wavelet coefficients
  (8) $$D_{\alpha j}=\frac{1}{n}\sum_{i=1}^{n}{\left({\text{y}}_{\text{i}}-\bar{\text{y}}\right)}^{2},D_{\theta j}=\frac{1}{n}\sum_{i=1}^{n}{\left({\text{y}}_{\text{i}}-\bar{\text{y}}\right)}^{2}\;(j=1,2,3,4)$$

where *j* represents the FC5, FC6, C3, and C4 channels; *i* denotes the wavelet coefficient of the alpha and theta rhythms; *n* denotes the total number of wavelet coefficients in each rhythm; *P*_α*j*_ and *P*_θ*j*_ are the energy of the wavelet coefficients of the alpha and theta rhythms from each channel, respectively; *D*_α*j*_ and *D*_θ*j*_ are the variance of the wavelet coefficients of the alpha and theta rhythms from each channel, respectively.

The feature set of the synchronous brain responses induced by facial expressions was computed for four channels and then concatenated to form a 1^*^16-dimensional feature vector *W*, which is described by:

(9) $$\begin{array}{r} W={\left[{\text{P}}_{\alpha j}{\text{P}}_{\theta j}D_{\alpha j}D_{\theta j}\right]}^{\text{T}} \end{array}$$

#### Classification

It is well known that the BPNN classifier provides good performances when classifying non-linear, self-adaptive and self-learning feature sets (Jiao et al., 2010). Thus, BPNN was chosen for EEG signal classification.

The BPNN performance is affected by three factors, training datasets, learning algorithms and network design. In this study, the training datasets were the WT coefficients from the EEG signals for each of the 4 facial expressions. Based on previous studies of training methods, a gradient descent with momentum algorithm was used for the learning stage because of its high convergence rate and short learning time.

The network design includes two steps. The first step is forming the network structure, where the input layer depends on the results of feature extraction, and the output layer is denoted by the number of signal types. No unified standard is available for the selection of hidden layer nodes, which are typically determined by the assessing of overall accuracy. Since the input of BPNN is the feature set of each trial, which was composed of 4 WT coefficients from 4 channels, the corresponding input layer of BPNN had 16 nodes. Due to the 2-DOFs prosthesis used in this study, the output layer had 2 nodes flagging the results [(0, 0) for Furrowing Brow, (0, 1) for Raising Brow, (1, 0) for Left Smirking, (1, 1) for Right Smirking]. Thus, a 3-layer BPNN model with 1 hidden layer was constructed. The hidden layer with 20 nodes showed the optimal performance. The structure of designed BPNN is depicts in the Figure 3B.

The next step in the BPNN design is determining the learning parameters, which include the values of the network weighted matrices, the learning rate and the error threshold. All layers of BPNN were connected together with the weights matrices. The value of weights must be normalized to small random numbers because the network may be saturated by large weighted values. The learning rate represents the rate of network learning, and the best values range from 0.1 to 0.9 (Omaima, 2010). The error threshold is the criterion used to evaluate the learning rate. The whole process for BPNN training is as follows:

1. Initial assessments of the network weighted matrices, learning rate and error threshold of the proposed BPNN.
2. In the beginning, set *k* = 1 and error *e* = 0. Obtain the feature vector *X*_*k*_ from the datasets, and feed it to the input layer, *k* = 1, 2, 3….*m*.
3. In normal propagation, calculate the outputs of hidden layer *b*_*in*_.
4. In normal propagation, calculate the outputs of the output layer *b*_0*ut*_.
5. Calculate the errors by subtracting the actual output from the desired output.
6. In backward propagation, adjust the network weighted matrices *W*_*in*_ and *W*_*out*_ based on the errors.
7. If *k* < *m*, then set *k* = *k*+1 and go to the Step 2; otherwise, compare the errors. If the errors < the threshold error, stop training; otherwise, go to Step 2.

To detect the robustness of the proposed BPNN and prevent an over-fitting problem, 5-fold cross-validation was used to investigate the classification accuracy, and each subject's data were used to train his/her own classifier. The offline dataset was randomly divided into 5 equal-sized subsets. The cross validation was repeated four times. During each validation, four subsets of data were used for training, and one was used for testing.

#### Statistical Analysis

According to the statistical theory, the choice of the sample size was depend on the three parameters: the expected effect size, the desired statistical power (1-β) and the significance level (α) (Desu and Raghavarao, 1990). Moreover, Cohen's *f* is one of the most widely used effect size measures in one way analysis of variance (ANOVA) (Cohen, 1988; Lakens, 2013). In this study, the desired statistical power was set to 0.8 (1-β = 0.8), the level of significance is 0.05 (α = 0.05) and the desired effect size is 0.9 (*f* = 0.9). Under this given condition, the estimated sample size is 18 subjects using statistical software G*Power.

One-way ANOVA was conducted to assess differences in the SampEn values between IMFs and the feature sets of four facial expressions. The recognition results from two different analyses of channel and feature comparisons were assessed using one-way ANOVA and the corresponding actual effect sizes of Cohen's *f* were also computed. The homogeneity of variance analysis was calculated to ensure the data is satisfies the assumptions of analysis of variance. Moreover, the Greenhouse-Geisser correction was applied for *p* value adjustments.

## Results

To validate the effectiveness of our proposed FE-BCI system for controlling a prosthesis, two experiments were conducted. The purpose of the offline experiment was to investigate the validity and reliability of the proposed system. During the online experiment, a 2-DOFs prosthesis was controlled using the FE-BCI system.

### Offline Experiment Analysis

**Figure 4:**
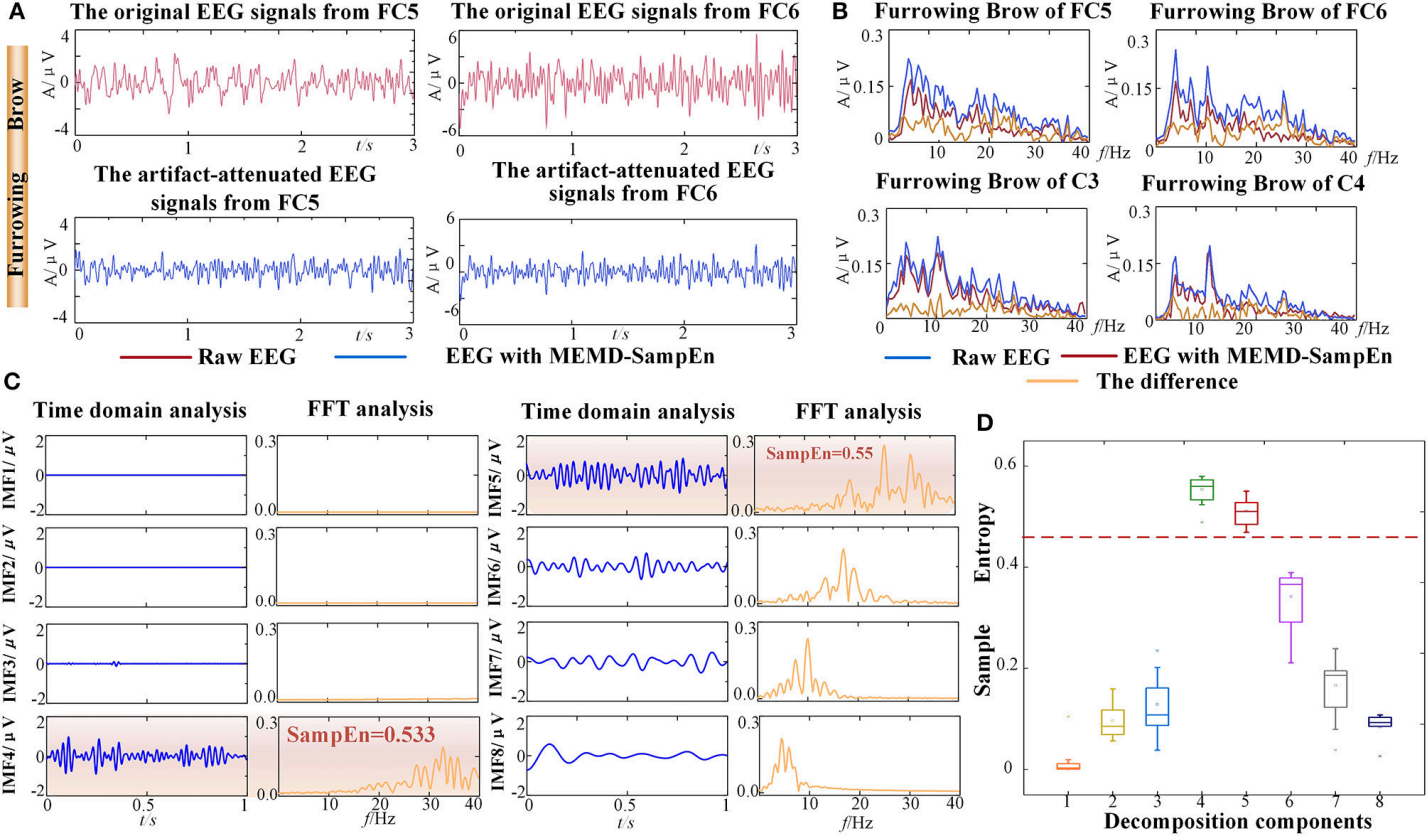
Results of EMG artifacts removal from one representative subject (S5). **(A)** The time series comparison associated with the grand average Furrowing Brow expression in FC5 and FC6. **(B)** The FFT comparison associated with the grand average Furrowing Brow expression in FC5, FC6, C3, and C4. The curves represented the original EEG signals, the artifact-attenuated EEG signals and the differences. **(C)** Time domain and frequency domain analyses of the decomposition components from the NA-MEMD with the average Furrowing Brow expression in FC5. The EMG artifacts are highlighted by an orange rectangle. **(D)** Statistical results of the average SampEn values from first eight IMF components. Dotted line is the threshold of 0.45.

To demonstrate the validity and reliability of FE-BCI system, the average offline data from subject S5 was analyzed. The other subjects showed similar results. To verify the performance of EMG artifacts removed by NA-MEMD combined with SampEn, the comparisons between the original EEG signals and the artifact-attenuated EEG signals were conducted associated with the grand average Furrowing Brow in FC5 and FC6. In time domain analysis, it is evident that the variability of the artifact-attenuated EEG signals was significantly alleviated, as seen in Figure 4A. In frequency domain analysis, the average frequency spectra were further analyzed using a fast Fourier transform (FFT). The low-frequency band (5-15 Hz) decreased slightly, while the high-frequency band (>15 Hz) dropped drastically after NA-MEMD, as seen in Figure 4B. To better observe the effectiveness of EMG artifacts removal, the first eight IMF components associated with the grand average FB in FC5 were reserved for further analysis, which are shown in Figure 4C. In Figure 4C, the first and third column presented the time-domain characteristics and the second and fourth column presented the frequency-domain analysis using FFT. It is observed that the frequency bands higher than 30 Hz were mainly located in the components of IMF 4 - IMF 5 and their SampEn values were significantly higher than 0.45. Considering the characteristics of EMG artifacts and the set threshold value of 0.45 in this study, the 4th−5th IMF components were marked as EMG artifacts, and hence discarded. Additionally, the statistical results from the SampEn, which included four select channels in four facial expressions, from the first eight components were calculated and then illustrated in Figure 4D. All the SampEn values for each IMF component showed good statistical properties, and the values of the 4th-5th IMF components were much higher than the others. It was also discovered that similar values of the IMFs for the four expressions. The result of one-way ANOVA demonstrated that there was a significant difference between IMF component (*p* < 0.05), especially between the 4th-5th IMFs and the other components. Both of the SampEn values and the frequency ranges demonstrated that these components were related to EMG artifacts and could be discarded.

Hence, these experimental results demonstrated that most of the EMG artifacts were successfully removed while brain activity were well preserved during the FE-BCI system working.

**Figure 5:**
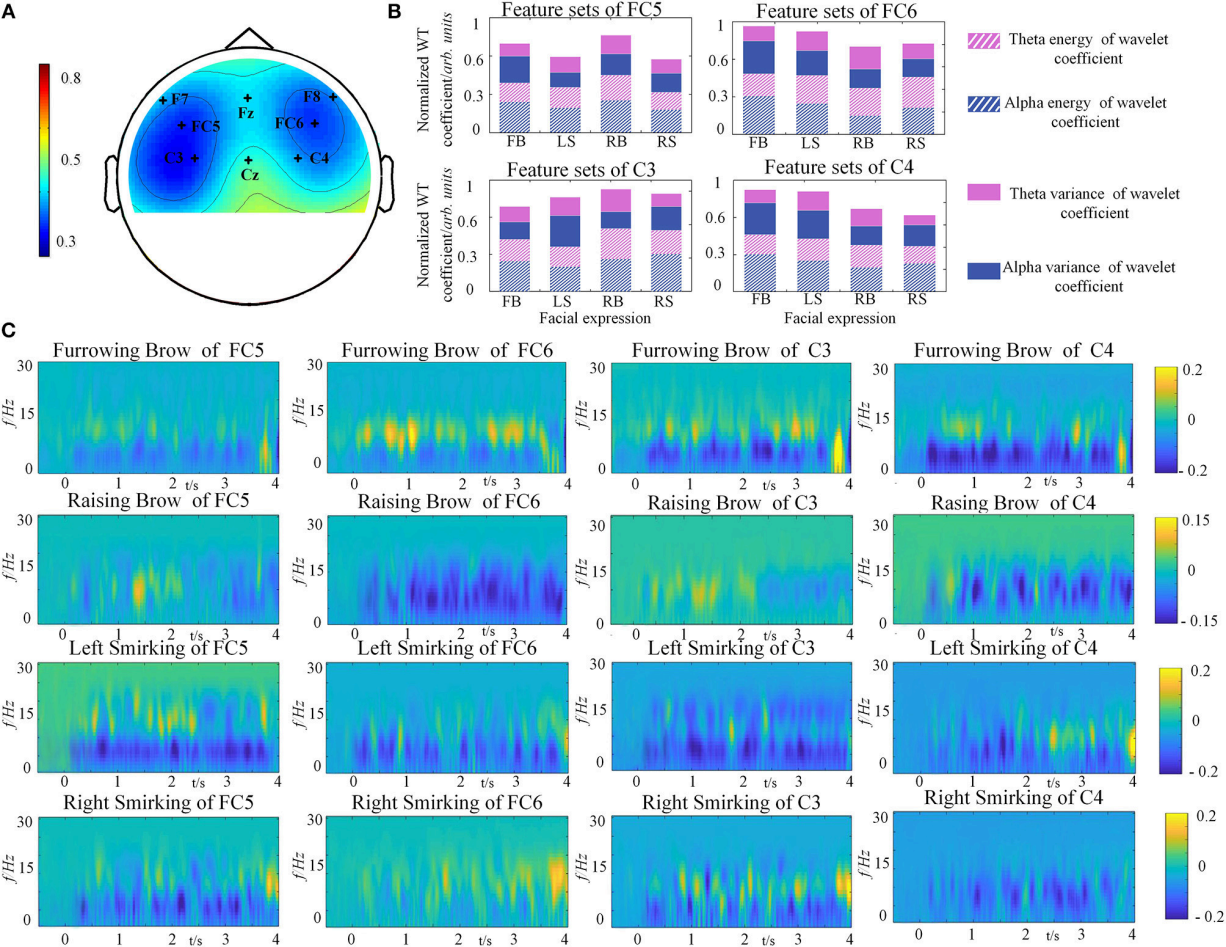
Grand average results from one representative subject (S5). **(A)** Topographic map of the correlation coefficients of four facial expressions' relations. The degree of correlation differences is depicted by the colored scale. **(B)** The feature sets of four facial expressions from C3, C4, FC5, and FC6. **(C)** The power distributions of the average four facial expression from FC5, FC6, C3, and C4.

To investigate the discriminative ability among different facial expressions, the global correlation coefficients between any two specific facial expressions at each channel were calculated. In statistics, the high correlation values indicate that the brain responses yielded by different tasks are similar to each other (Hsu, 2015). Hence, discrimination is reversed with correlations. For accurate descriptions, topographic maps were constructed to represent the grand average correlations among all channels for the 4 types of facial expressions, as illustrated in Figure 5A. The results of one-way ANOVA indicated that the correlations were significantly different on 8 channels, and a rare connection was found between the prefrontal and motor cortices, especially at FC5, FC6, C3 and C4 (*p* < 0.05). These results demonstrated that the four selected electrodes (FC5, FC6, C3, and C4) in the prefrontal and motor cortices contribute significantly to distinguish different facial expression. This finding is also consistent with the physiological mechanisms of facial expressions (Keltner et al., 2003; Kilts et al., 2003).

The normalized WT coefficients features of facial expressions for each electrode were depicted in Figure 5B. It can be clearly seen that the brain activity of the four facial expressions showed different characteristics of the selected rhythms for each channel. In the case of C3 for instance, the power of alpha rhythm was smaller in Left Smirking, while the variance of theta rhythm was higher than the Furrowing Brow. It is quite evident that there are obvious power changes existed between alpha and theta rhythm under C3, C4, FC5, and FC6. The one-way ANOVA analyses for the feature sets of four facial expressions were conducted. The result indicated that the significant differences were observed among different feature sets of four facial expressions, whereas not such differences were found among subjects (*p* < 0.05).

In order to understand the brain responses to the FE-BCI paradigm comprehensively, EEG signals evoked by four facial expressions were converted into time-frequency characteristics using a short-time Fourier transform (STFT), as shown in Figure 5C. Here, we used the EEG signals from the S5 as an example. The other subjects showed similar responses. Figure 5C shows that the stable change of power from the EEG signals was dominant at a range of 4–16 Hz among four facial expressions, where the alpha and theta rhythms were located after a latency stage. By comparing to the power in resting state, significant energy changes were recorded during the four expressions. Using Furrowing Brow as an example, the power of alpha energy increased while the power of theta energy decreased over the prefrontal and motor cortices, respectively. Although the energy change covered these cortexes, the right hemispheres (FC6 and C4) was more dominant than the left hemispheres (FC5 and C3) during the Furrowing Brow. Interestingly, significant alpha energy changes occurred over the motor cortex (C3 and C4) during both Left and Right smirking. In more details, there is a contralateral power increase of alpha rhythm and an ipsilateral power decrease of the same rhythm for the expression of left and right smirking. These performances are consistent with previous research (Paradiso et al., 2005). Most importantly, these results further verified that the prefrontal and motor cortices play an important role in the classification of different facial expressions, and the power variations in alpha and theta rhythms occurred during the expression presentation.

Therefore, the above findings confirmed that facial expressions could induce discriminable brain responses at the corresponding representation areas. Most importantly, these results indicated that the brain activity from the prefrontal and motor cortices was sufficient for categorization.

**Figure 6:**
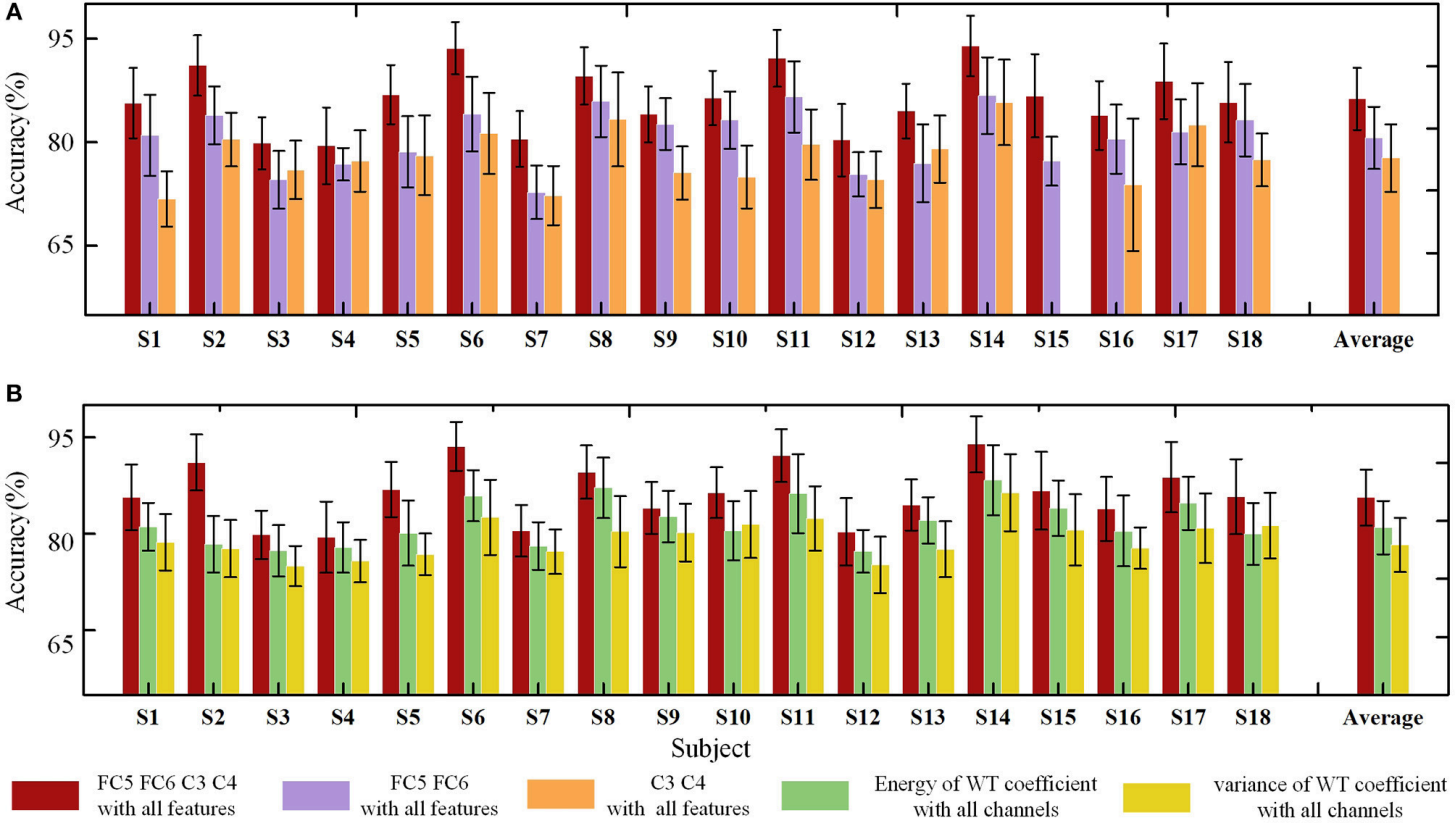
Offline classification accuracies of standard deviations using two analyses for each subject. **(A)** Offline accuracies of three channel conditions: FC5, FC6, C3, C4 vs. FC5, FC6 vs. C3, C4. The red bar indicates the accuracies based on the proposed method, the violet bar indicates the accuracies based on the FC5 and FC6 channels, and the orange bar denotes the accuracies based on the C3 and C4 channels. **(B)** Offline accuracies of three feature set conditions: energy of the WT coefficient vs variance of the WT coefficient vs the combination of these features. The red bar indicates the accuracies based on the proposed method, the green bar indicates the accuracies based on the energy of the WT coefficient, and the yellow bar denotes the accuracies based on the variance of the WT coefficient.

To determine the efficiency of the selected methods in the FE-BCI system, the offline classification accuracy was estimated using two different analyses, channel comparisons (FC5, FC6, C3, C4 vs. FC5, FC6 vs. C3, C4) and feature comparisons (energy of the WT coefficient vs. variance of the WT coefficient vs the combination of these features). Figure 6 summarizes the grand average offline accuracy obtained for all subjects. The final accuracy was the average value of the four runs of the 5-fold cross validation. As seen in Figure 6A, all subjects achieved performance higher than 72.69% in the three conditions; all subjects showed performances that were significantly higher than the chance level (the chance level was 29.58% for each subject and 26.09% for the group). The performance of the proposed channel selection set showed the highest classification rate for all subjects. In addition, the grand average accuracy across all subjects was 81.28 ± 4.5% in the selected channel condition, and the highest value was 88.94 ± 4.37% from S14. It was clear that the brain responses of the motor cortex exhibited the lowest accuracy. The statistical analysis was also used to assess the performance of two different comparisons (channel and feature comparisons), which include the significant difference and the effect size analysis. During the channel comparisons, the significant differences and large effect were found among the three conditions using one-way ANOVA (*p* < 0.05, *f* = 0.93). Moreover, the efficiency of the feature set selection in the FE-BCI system was also estimated under three different conditions: wavelet energy-only, wavelet variance-only and the combination of these features. The respective grand average accuracies were 76.80 ± 4.40, 74.33 ± 4.55, and 81.28 ± 4.5%. In addition, the performance of the combined feature set showed the highest classification rate for all subjects. One-way ANOVA was used to compare the performances of the three feature set conditions (*p* < 0.05, *f* = 0.86). These results validated that the proposed approach of computing the temporal feature set was efficient and that the combined set of WT coefficients was the most valuable condition for detecting the characteristics of facial expressions.

Overall, the offline analysis result proved that the FE-BCI system was capable of practical applications.

### Online Analysis

The offline experiment analyses demonstrated the effectiveness of the proposed FE-BCI system. Hence, our online experiment used the proposed system to control a 2-DOFs prosthesis with four discrete gestures: Hand Opening (HO) by raising of the brow, Hand Closing (HC) by furrowing of the brow, Wrist Rotation Right (WRR) by left-side smirking, and Wrist Rotation Left (WRL) by right-side smirking.

During the online experiment, each subject performed ten sessions. In each session, the subjects were instructed to finish a complete prosthesis movement, i.e., HO-HC-WRR-WRL-HO-HC, which imitated a disabled person drinking water.

**Figure 7:**
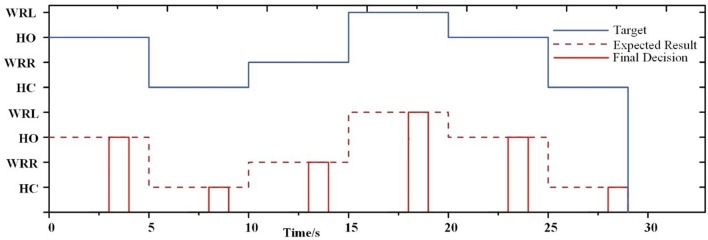
Example of the online experiment for each session in one representative subject (S5).

As seen in Figure 7, it presents an example of a single session from subject S5 where a decision was generated at the end of 4 s. Before a decision was generated, the prosthesis remained in the previous gesture. This example shows the feasibility of the FE-BCI for prosthesis control.

**Figure 8:**
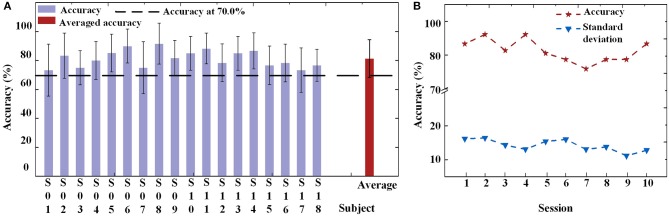
Online performance of prosthesis control. **(A)** Classification accuracies and standard deviations of each subject and the averaged accuracy. Dotted line is the criterion of 70%. **(B)** Classification accuracies and standard deviations of each session.

For the online experiment, all offline data were applied to train the BPNN classifier. Each subject had their own classifier. The average accuracy for each subject across all sessions is shown in Figure 8A. When using the FE-BCI system to control the prosthesis, the subjects achieved an overall accuracy of 81.31 ± 5.82% across all subjects and sessions, which was significantly higher than the chance level (the chance level was 35%). Most of the subjects showed good performance, with the exception of S1. It is difficult to identify reasons for this subject's low accuracy, although the most likely causes might be mental fatigue and inattention. Notably, S6 and S11 showed good performances for both offline and online experiments, with accuracies up to 87.90 ± 11.65 and 88.34 ± 10.67%. The result of the one-way ANOVA for the sessions indicated that there was no significant differences in the accuracy of prosthesis controlled by FE-BCI system during the experiment (*p* > 0.05). To further analyse the effects of fatigue over the whole experiment, the accuracy of each session was calculated for each of the eighteen subjects, and the average accuracy across all subjects for each session is presented in Figure 8B. The averaged accuracy for the grasping task increased from 83.34 ± 16.16% in the first session to 85.18 ± 12.63% in the fourth session and remained at an average of 80.25 ± 12.56% across the last three sessions. There were no significant fluctuations were found over the time in terms of average accuracy and its variances (*p* > 0.05). These results demonstrated that the efficiency of the FE-BCI was stable and suitable for controlling prostheses.

## Discussion

The aim of this study was to assess the efficiency of a novel FE-BCI system and the feasibility of controlling a prosthesis with this system. Four facial expressions were selected as targets for emerging brain responses, and NA-MEMD + SampEn combined with a WT+ BPNN was selected as the processing algorithm. The experimental results support the hypotheses that the FE-BCI system could yield good performance and could feasibly control a prosthesis.

### The Significance of Facial Expression Mechanisms

In this study, we hypothesized that the prefrontal and motor cortices are responsible for facial expression presentation. It is widely acknowledged that the processing of facial expression in the cortical regions of the brain is complicated, involving diverse neural pathways, different information sources and expressions. It is difficult to show that the synchronous brain responses involved in facial expressions are unique to the prefrontal or motor cortices, as these responses can be found elsewhere in brain, to varying degrees and in various modalities, such as the limbic system (Miller and Cohen, 2001). However, there is no doubt that the prefrontal cortex combined with the motor cortex provides critical contributions to facial expression processing. Neurophysiological studies have explained several detailed properties of the prefrontal cortex, including that prefrontal areas coordinate neural activity from other cortices (Stuss and Benson, 1984; Morecraft et al., 2001). In addition, assessments of the function of the motor cortex in recent studies also provide convincing explanations for the relationship between activity in this area and the representation of facial expressions (Marinkovic et al., 2000; Keltner et al., 2003; Mandal and Awasthi, 2015). Unfortunately, the typical ERD/ERS was not found and the energy changes in alpha and theta rhythms still existed. One of the possible reasons for this phenomenon is that the movements of facial expressions are far away from the body movement, including the accurate projection area in sensorimotor cortex, the nervous system transmissions, motor neuron activity generation, facial nerve transmissions and realizations.

### The Performance of the FE-BCI System

Even though the neural mechanisms of facial expressions are not yet fully understood, different facial expressions induce distinct brain responses in the prefrontal and motor cortices. One of the major problems in BCI systems is the artifact contamination. Due to each neurological phenomenon has its unique motivation as well as spatiotemporal characteristics. Hence, this fact should be taken into consideration when addressing the presence of artifacts. In the FE-BCI system, the common artifacts influencing the quality of EEG signals are facial EMGs, which are sensitive to varieties of facial expression processes. Therefore, our study focused on EMG artifacts removal. The results of (Fatourechi et al., 2007; Mowla et al., 2015) showed that the NA-MEMD algorithm was able to alleviate the effects of mode mixing and is more suitable for attenuating EMG artifacts in a few-channel BCI system. In information theory, SampEn represents the complexity of a signal. Because the randomness of EMG artifacts is stronger than that of EEG signals, the value of the EMG artifacts is much higher than that of EEG signals (Teng et al., 2014). Hence, NA-MEMD combined with SampEn is appropriate to remove EMG artifacts in the facial expression paradigm (Figure 4). Moreover, since the EMG activity generally consist of high-frequency components and have some overlapping frequency with beta rhythm (Pfurtscheller et al., 2000b). Hence, the FE-BCI system that uses multiple neurological phenomena from the theta and alpha bands may become more robust to present the characteristics of different facial expressions. To better verify the effectiveness of the proposed system, the characteristics of four specific facial expressions were carefully investigated. Topographic maps of correlation coefficients were used to evaluate the discriminative ability of the FE-BCI system and the efficiency of the selected electrodes (Figure 5A). A series of time-frequency analyses based on STFTs and classification accuracies confirmed that the alpha and theta rhythms played an important role in facial expression descriptions (Figures 5B,C). These analyses are consistent with previous reports of facial expression processing mechanisms (Marinkovic et al., 2000; Keltner et al., 2003; Paradiso et al., 2005; Mandal and Awasthi, 2015; Ross et al., 2016). Most importantly, the analysis of the EEG characteristics denoted by facial expressions further demonstrated the efficiency of the selected characteristics. Customizing the features used for classification is important in a BCI system.

The recognition accuracy assessments also demonstrated the efficiency of the FE-BCI system. We assessed 240 trials for 4 targets classified in the offline experiment and a total of 60 trials in the online test. Theoretically, the chance level performances for these experiments were 29.58 and 35%, respectively. Specifically, the offline accuracy across 18 subjects was 81.28 ± 4.5%, and the average online accuracy was 81.31 ± 5.82% (Figures 6, 8), both of which were significantly higher than the empirical chance level (Müller-Putz et al., 2008; Combrisson and Jerbi, 2015). Among all the subjects, S6 and S11 obtained significantly high accuracies that exceed 87%. In BCI practical applications, the accuracy of a BCI system is defined as the ability to avoid unintended and false communications and is required to be above 70% (Kübler et al., 2001). This criterion has also been used in most of the previous literature (Brunner et al., 2010; Hwang et al., 2013). In addition, Cohen has provided the benchmarks of effect size conventions in ANOVA for accessing experimental effects into small (*f* = 0.1), medium (*f* = 0.25), and large effect (*f* = 0.40). Based on these benchmarks, the actual effect size of our experiment was higher than o.4, which also demonstrated good effect in the practical significance as well as the stability of the proposed system. Hence, these recognition results indicated that the developed BCI system could be used for prosthesis control. It is also demonstrated that the signal processing approach in the proposed BCI system is effective at classifying users' intentions. When considering these results together, this study provides convincing evidence of the validity and reliability of the FE-BCI system and the feasibility of its use in daily life.

### Comparison With Other FE-BCI System and Brain Controlled Prostheses Methods

As briefly introduced in section Introduction, the current brain controlled prostheses methods are mainly focused on MI-BCI system, P300-BCI and SSVEP-BCI system (Pfurtscheller et al., 2010; Li et al., 2017; Samuel et al., 2017a). Pfurtscheller et al. were the first to use a MI-BCI system to help a tetraplegia patient operating his prosthesis. Mall used a MI-BCI system in monkeys to control a mechanized arm. The MI-BCI system do not easily facilitate the execution of prostheses tasks due to the long training period, the number of available commands and the portability of the BCI system. Specifically, the available commands of most MI-BCI systems are limited to three, and the recognition accuracy is not entirely satisfactory (Pfurtscheller et al., 2000b). Even though classification has been significantly improved by SSVEP-BCIs and P300-BCIs, long time stimulation by normal stimulus easily lead to visual fatigue and increases risk of triggering a photosensitive epileptic seizure for subject.

To improve the limitation between BCI performance and its stimulus reliance, a novel BCI system based on facial expression have been developed. Most studies related to FE-BCI system usually focusing on face-based video or face-based image induced systems (Bakardjian et al., 2011; Jin et al., 2012; Vinding et al., 2014). For example, a study by Jin and colleagues proposed a FE-BCI system with an emerging stimulus, which used the images of facial expressions to evoke ERPs (Jin et al., 2014). This facial expression paradigm yielded better performance than the canonical visual stimulus approach. In another study, Kashihara used a face-based video paradigm to reduce subject fatigue and enhance visual attention (Kashihara, 2014). Although these visual attention-based FE-BCI systems improved the responses of evoked potentials, the obstacle between the good performance of BCI system and the independence on stimulus are not completely solved. This phenomenon could further limits the mobility of FE-BCIs in daily life applications. To overcome this limitation, we proposed a novel FE-BCI system based on real facial expression, which does not required an additional hardware device to present stimulus. In contrast to previous studies, we recorded brain response triggered by real facial expressions, which yielded effective performance. More recently, Chin et al. reported a similar method based on real facial expressions system (Chin et al., 2008). However, the system was not a complete BCI system due to record EEG and EMG signals. As EEG signals are very sensitive to EMG signals, the contributions of each kind of signal was not clear. Additionally, the selection of the channels was not investigated. In contrast, our study only used four EEG electrode concerning the mechanism of facial expression. Hence, our research can provide another option for disabled people in addition to the traditional BCI systems (MI-BCI, SSVEP-BCI, P300-BCI). The benefits of using a FE-BCI system may be its high classification accuracy and high mobility. A system that can be used without extensive training and additional hardware is appealing since it requires less initial effort on the part of both the subject and the system operator.

For daily-life application, decoding the EEG signals from a few channels is challenging for BCI systems, and most previous studies have used sophisticated electrode selections when controlling prostheses (Fatourechi et al., 2008; Stan et al., 2015; Vidaurre et al., 2016). In our study, only four electrodes were used. The use of the wireless EEG acquisition system Neuracle rather than SynAmps 2 may motivate the use of the FE-BCI in daily life environments. Minimizing the number of required recording electrodes is vital for any EEG-based command system to be practical for everyday use. The FE-BCI system had a low cost and used a simple electrical montage compared with other brain-controlled prostheses. From the user's perspective, systems with simple electrodes and lower costs are more user-friendly style. These metrics make FE-BCI-controlled prostheses practical for disabled users.

### The Limitations of the Study and Further Work

Despite the aforementioned advantages of FE-BCI, several limitations should be taken into account. One of the limitation is that only healthy subjects were studied. There is no doubt that the main motivation for our study is to provide a novel facial expression based BCI system for controlling a prosthesis, which solve the obstacle between the good performance of BCI system and the independence on stimulus. The results presented in our study were investigated the feasibility of FE-BCI usage in disabled people and contribute to a better understanding of the performance of the proposed system. To the best of our knowledge, there are no comparative works reported that the significant differences existed between the healthy and amputee during the facial expressions. One of the possible reasons is that the mechanism of facial expressions are far away from the mechanism of hand movement, including the accurate projection area in sensorimotor cortex, the nervous system transmissions et al. (Miller and Cohen, 2001; Mandal and Awasthi, 2015). Even if the patient is amputated, his/her mechanism of facial expression may not be influenced significantly. Thus, we speculate that the proposed system could be effective for disabled persons. Further study will involve disabled people using FE-BCI system to control a prosthesis. Another limitation of this study is the samples size that only contained 18 subjects and there was a gender imbalance among subjects. Even though the size of experimental group was not large enough, the number was sufficient to demonstrate the effectiveness of proposed method and highlight some significant statistical evidences. The offline accuracy across 18 subjects was 81.28 ± 4.5%, and the average online accuracy was 81.31 ± 5.82%, both of which were significantly higher than the empirical chance level. Moreover, no significant differences was found between male and female subjects in the experimental results. Thus, large sample and a balance between male and female subjects are desired to fully evaluate the robustness of the proposed system. Furthermore, it is important to have beginning and stopping commands in brain-controlled prostheses, and the time required to remove artifacts also needs improvements. In our future work, another BCI paradigm will be added to build a hybrid BCI system for an “idle states” detection.

## Conclusion

This paper presented a novel BCI system based on facial expressions that was used to control a prosthesis. The synchronous brain responses from four types of facial expressions were used to control a 2-DOFs prosthesis. Both online and offline experiments were conducted. The experimental results demonstrate the effectiveness of our method and system. Thus, our proposed FE-BCI system can provide another option for patients suffering from amputations.

## Author Contributions

RL, XZ, and CL designed the study. HL and ZL performed the experiment research. RL, XZ, and CL performed the theoretical analysis. RL, HL, WS, and RO analyzed the data, prepared the figures and tables, and wrote the manuscript.

### Conflict of Interest Statement

The authors declare that the research was conducted in the absence of any commercial or financial relationships that could be construed as a potential conflict of interest.
